# Supplementary material for: A conformation-selective monoclonal antibody against a small molecule-stabilised signalling-deficient form of TNF
Source: Nat Commun. 2021 Jan 25;12:583. doi: 10.1038/s41467-020-20825-6 (PMC7835358; doi:10.1038/s41467-020-20825-6)
Supplement: Supplementary file 1 — Supplementary Information [file 41467_2020_20825_MOESM1_ESM.pdf]

A conformation-selective monoclonal antibody against a small molecule-stabilised signalling-deficient form of TNF

Lightwood et al.

Supplementary Figures

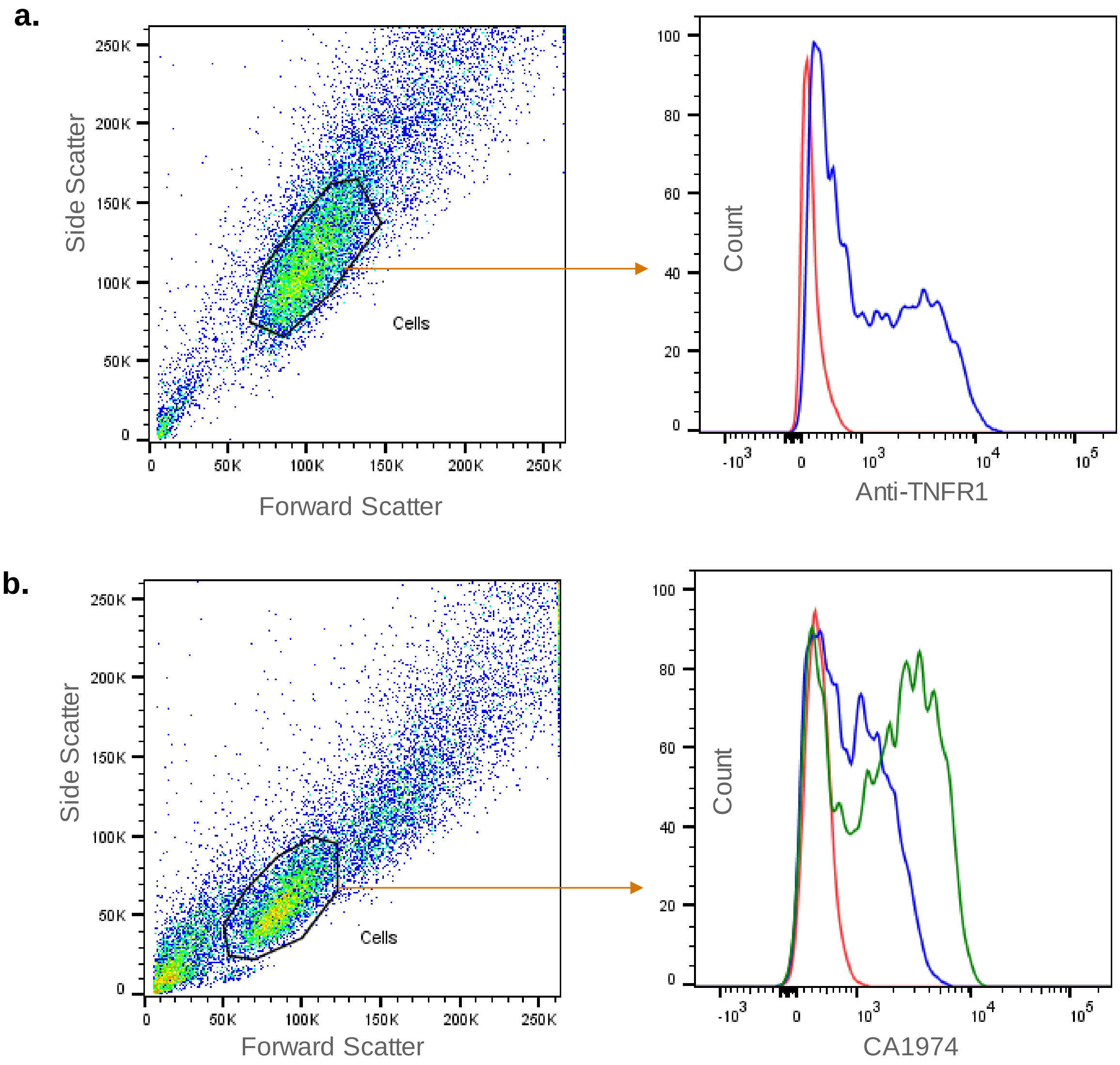

Supplementary figure 1 – Gating strategy used for CA1974 staining of TNFR1 expressing HEK-293 Jump-In cells

(a) Gating strategy to show human TNFR1 expression on HEK-293 Jump-In cells following treatment with doxycycline at 1µg/ml for 2.5 hours. Cells were incubated with anti-human TNFR1 monoclonal antibody at 10µg/ml (MAB225, R & D Systems) (blue) or mouse IgG1 isotype control (#026100, Invitrogen) (red) followed by Goat-anti-mouse-Alexa 488 secondary antibody (#A-1101, Molecular Probes) at 1:200 dilution. Count (*y*-axis) is normalised to mode.

(b) Gating strategy used to identify TNFR1-expressing HEK-293 Jump-In cells showing CA1974 staining of human TNF-**UCB-9260** complex shown in Figure 1b. Representative scatter profile shows 25ng/ml of TNF (blue). The TNF–DMSO control (red) and 250ng/ml TNF (green) were gated using the same strategy. Count (*y*-axis) normalised to mode. Source data are provided as a Source Data file.

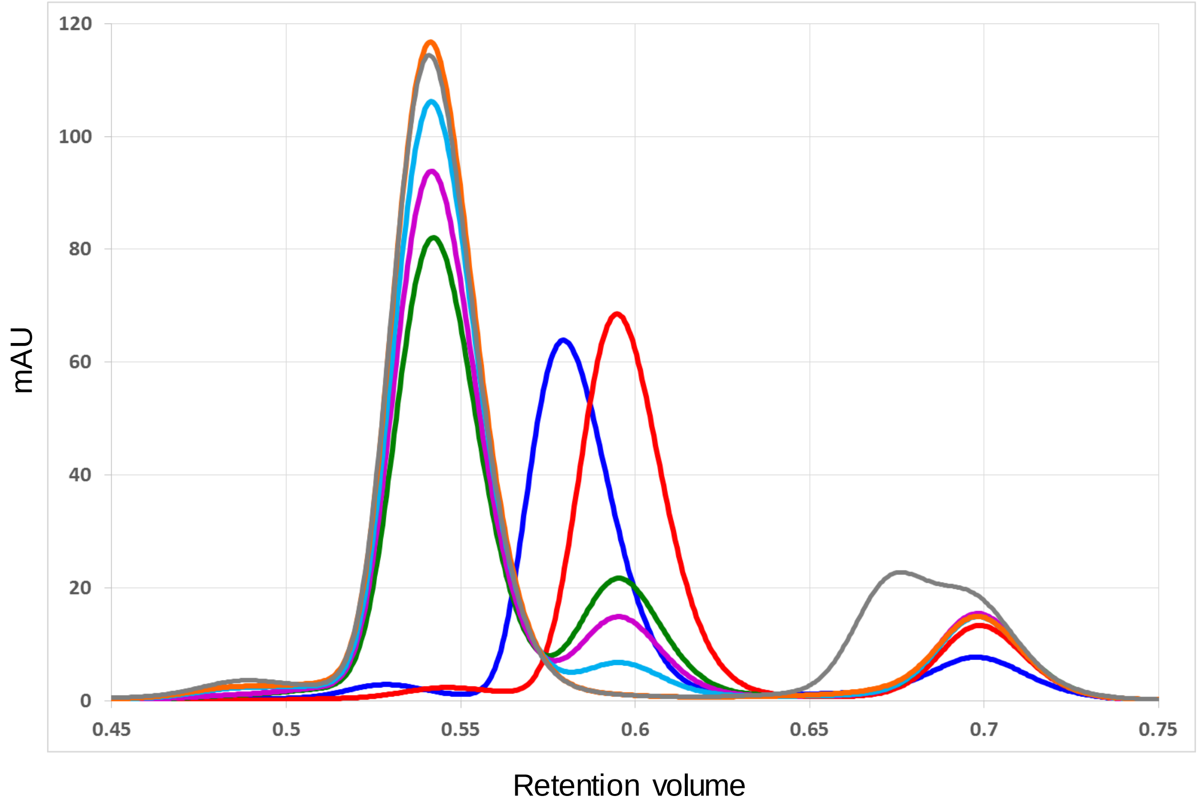

Supplementary figure 2 – Analytical size exclusion of CA1974 Fab-human TNF-UCB-8733-human TNFR1 complex

Human TNF bound to **UCB-8733** and 3.5 molar equivalents of TNFR1 was incubated with increasing molar ratios of CA1974 Fab. Human TNF without compound + 3.5x TNFR1 (blue), TNF + **UCB-8733** + 3.5x TNFR1 (red), and with increasing molar ratios of CA1974 Fab: 0.6x (green), 0.7x (magenta), 0.8x (cyan), 0.9x (orange) and 1.0x (grey). Source data are provided as a Source Data file.

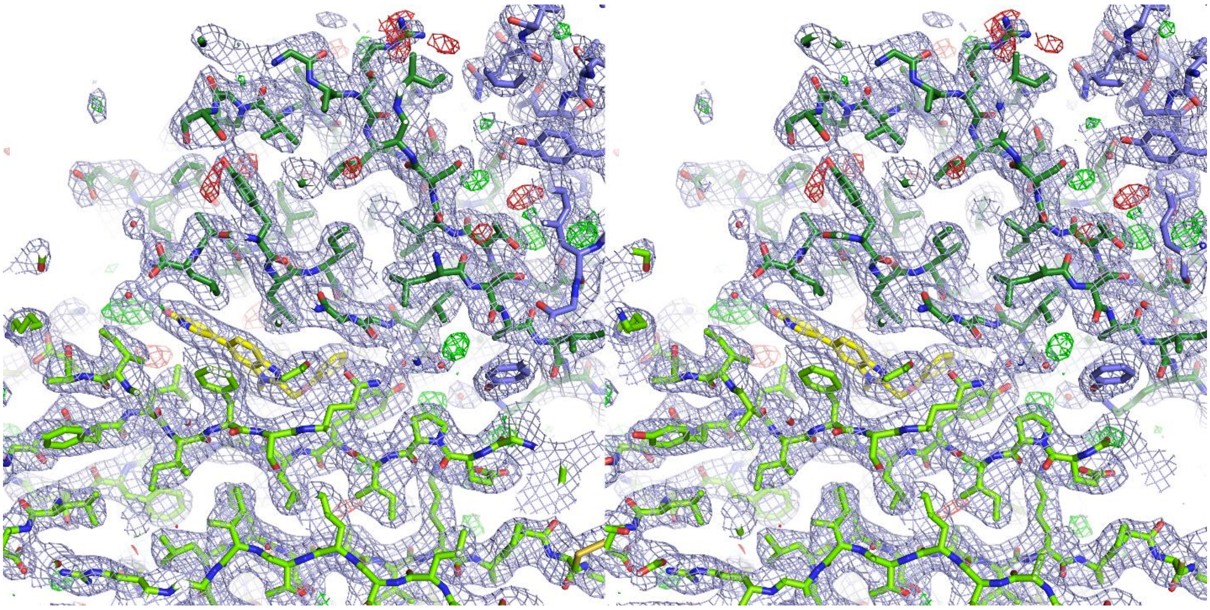

Supplementary figure 3 - Electron density for CA1974 Fab-human TNF-UCB-8733-human TNFR1 complex Trimeric human TNF (green sticks) with **UCB-8733** (yellow sticks) and CA1974 Fab (blue sticks), receptors not shown. 2Fo-Fc maps contoured at 1.0σ (blue mesh), Fo-Fc maps contoured at +/-3.0σ (green/red mesh).

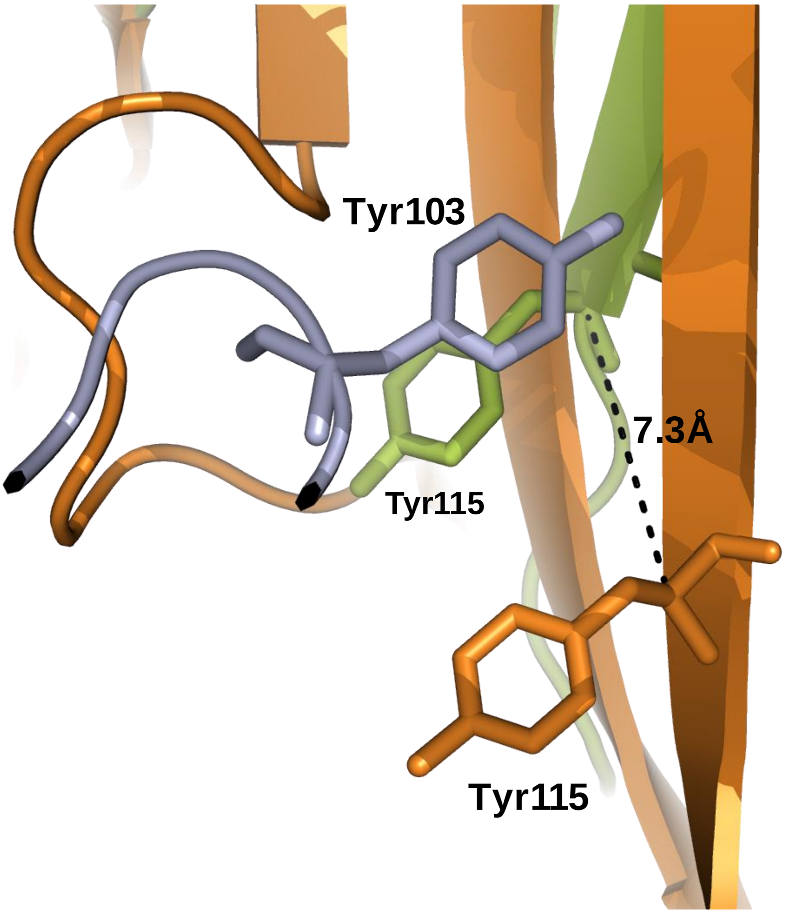

Supplementary figure 4 – Detail showing the relative positioning of Tyr115 on TNF monomer C (plus and minus compound) in relation to Tyr103 of CA1974 heavy chain CDR3

Detailed view of CA1974 heavy chain CDR3 (blue cartoon) showing Tyr103 (blue sticks) pi stacking with Tyr115 (green sticks) of the compound-bound TNF (green cartoon) and the relative position of Tyr115 (brown sticks) of the apo TNF (brown ribbon) (after aligned through monomer A). The degree of displacement of Tyr115 is indicated (black dashed line) and relevant amino acids are labelled.

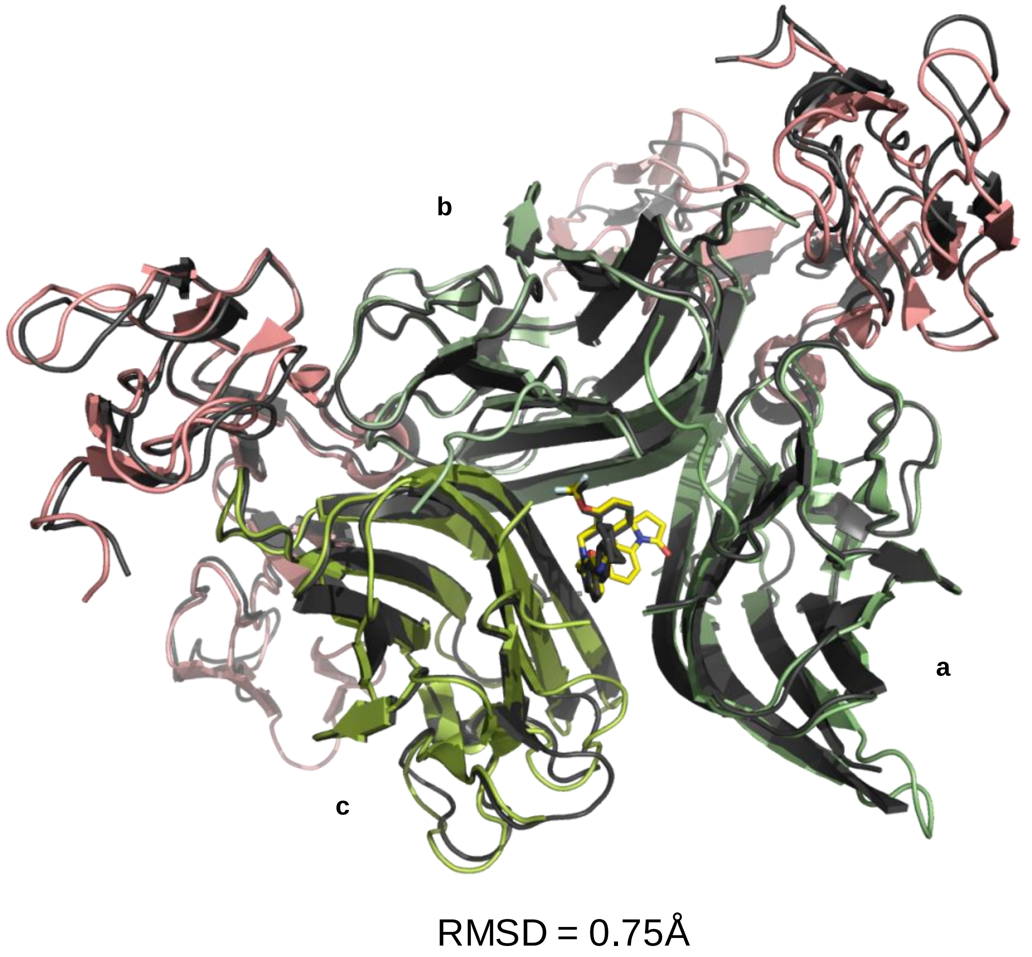

Supplementary figure 5 - Comparison of human TNF-human TNFR1 complex from the Fab CA1974 Fab structure with mouse TNF-human TNFR1 complex from PDB code: 7KP8

Overlay of mouse TNF-human TNFR1-**UCB-4433** structure (from McMillan et al.)[1] (PDB code: 7KP8) (grey ribbons) with the human TNF-human TNFR1-**UCB-8733**-CA1974 structure (Fab removed from a/c binding site clarity) (green ribbons) (PDB code 7KPB. Overlay and RMSD value confirms the similarity in the binding mode of human TNFR1 to the different species of TNF.

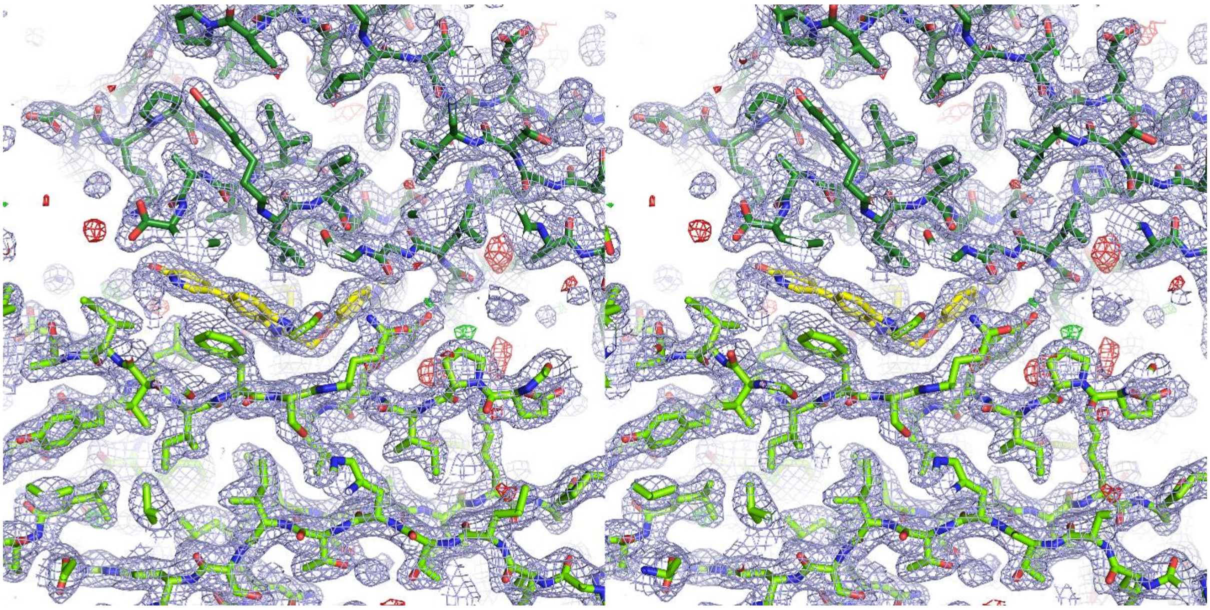

Supplementary Figure 6 - Electron density for human TNF bound with UCB-8733
Trimeric human TNF (green sticks) with **UCB-8733** (yellow sticks). 2Fo-Fc maps contoured at 1.0σ (blue mesh), Fo-Fc maps contoured at +/-3.0σ (green/red mesh).

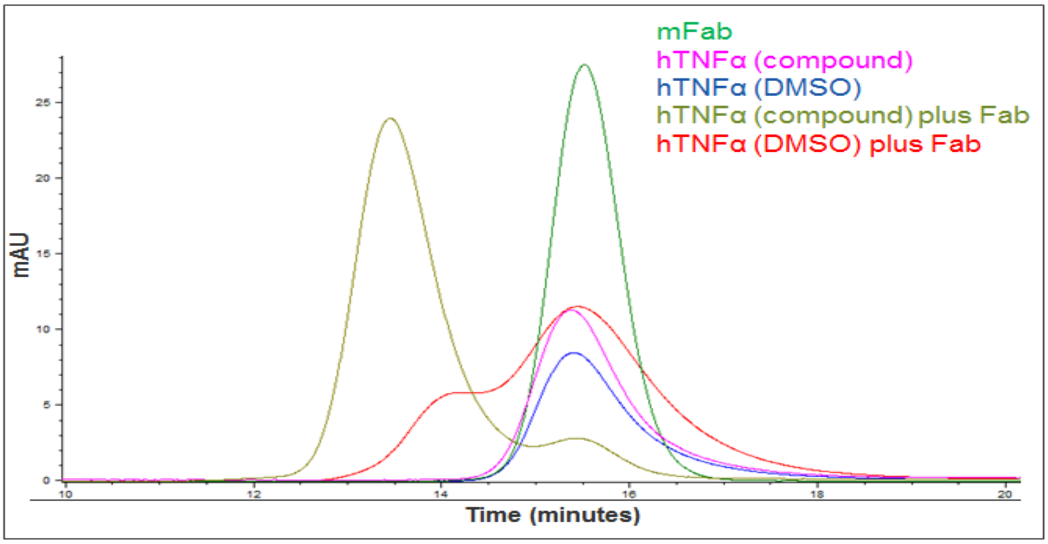

Supplementary figure 7 – Size exclusion (SE)-HPLC analysis of the CA1974 interaction with apo TNF
The Fab fragment of CA1974 was incubated at an equimolar ratio with either TNF-**UCB-9260** small molecule inhibitor complex (khaki green) or apo TNF (red) and then analysed by SE-HPLC. Fab alone (bright green), TNF-**UCB-9260** complex alone (pink) and apo TNF alone (blue) were used as controls in the assay. The elution profile suggests that a fraction of apo TNF interacts with CA1974 and produces a complex that elutes at a similar time point to the CA1974-TNF-UCB-9260 complex (which had been determined to be a 1:1 stoichiometry). Source data are provided as a Source Data file.

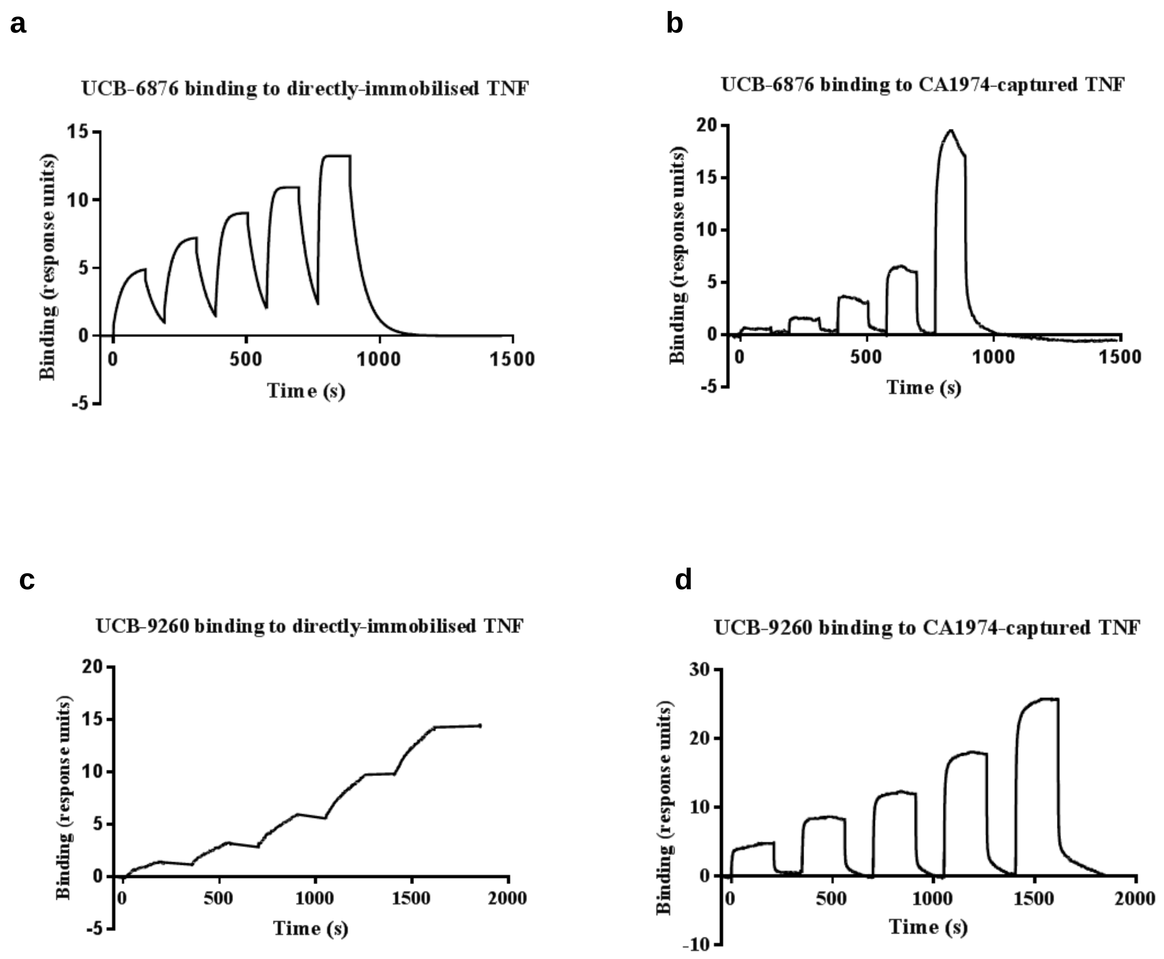

Supplementary figure 8 – Single cycle kinetics of UCB-6876 and UCB-9260 binding to human TNF directly immobilised or via capture with CA1974

For single cycle kinetics profiling of **UCB-6876 (Described in O'Connell et al., 2019)**[2] (a and b) and **UCB-9260** (c and d) to either directly immobilised TNF (a and c) or CA1974-captured TNF (b and d), surface plasmon resonance was performed at 25 °C using a BIAcore T200 (GE Healthcare). Human TNF was tethered onto flow-channel (Fc)2 (~2000 RU), CA1974 IgG (>10,000 response units) tethered onto Fc3,4 and Fc1 blank immobilisation on a CM5 Sensor Chip (GE Healthcare) via amine coupling chemistry. Human TNF was flowed over Fc4 to a capture level of ~2,000 RU. Compound **UCB-6876** was flowed over all four work cells in series (15.625 µM, 31.25 µM, 62.5 µM, 125 µM and 250 µM) at 30 µl/min. Compound **UCB-9260** was flowed over all four flow cells in series (1.875 µM, 3.75 µM, 7.5 µM, 15 µM and 30 µM) at 100ul/min. Double referenced background-subtracted binding curves were produced using the T200 Evaluation software (version 1.0) following standard procedures. As indicated, kinetics change from slow on-slow off with directly immobilised apo-TNF to fast on-fast off when captured via the conformation-selective antibody CA1974. This indicates that the antibody is able to stabilise an alternative, presumably open conformation which might facilitate the identification of new chemical matter which, under normal circumstances, is entropically disadvantaged. Source data are provided as a Source Data file.

Compound	Structure	ka ($M^{-1}s^{-1}$)	kd (s^{-1})	K_D (nM)
**UCB-9260**	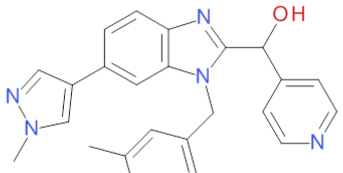	**3.17E+03**	**4.39E-05**	**13.8**
**UCB-8733**	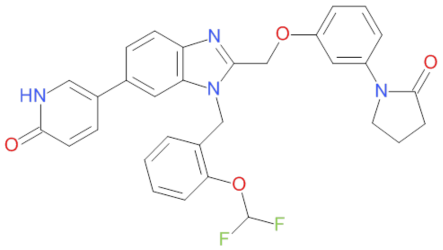	**4.52E+03**	**3.68E-05**	**8.1**

Supplementary figure 9 – structure and affinity information for UCB-9260 and UCB-8733
Structures of **UCB-9260** and **UCB-8733** are shown. Biacore was used to determine binding kinetics of small molecule inhibitors to human TNF. The ka, kd and K_D are shown. Data for **UCB-9260** is based on n=1, data for **UCB-8733** is based on n=3 independent experiments. Source data for **UCB-8733** is provided as a source data file. Data associated for **UCB-9260** is taken from O'Connell et al. (2019).[2] Source data are provided as a Source Data file.

Supplementary Tables

Residue	Movement Å	
	hTNF(UCB-8733) from Fab/receptor structure	hTNF(UCB-8733) from TNF/compound only structure
L75	8.3	8.6
T77	7.4	7.4
L94	7.5	7.6
I97	7.2	7.3
V123	7.5	7.6

Supplementary table 1 - Measurements showing the degree of movement in monomer A of TNFα captured by the compound UCB-8733 in the presence and absence of CA1974

An overlay of human TNFα with **UCB-8733** bound, taken from the compound only structure and from the structure with CA1974 bound, aligned through monomer C was used to measure the displacement of monomer A. The degree of displacement (in Å) of selected residues on monomer A are shown (measurements taken on the α-carbon atoms).

TNFa monomer A		
Human	Mouse	Cyno
T77	T76	T77
T79	T78	T79
I83	F82	I83
T89	E88	T89
K90	K89	K90
V91	V90	V91
N92	N91	N92
L93	L92	L93
L94	L93	L94
S95	S94	S95
A96	A95	A96
I97	V96	I97
K98	K97	K98
E135	E134	E135
I136	V135	I136
N137	N136	N137
R138	L137	L138

TNFa monomer C		
Human	Mouse	Cyno
L63	L63	L63
K65	K65	K65
Q67	Q67	Q67
P113	P112	P113
Y115	Y114	Y115
D143	D142	D143
A145	A144	A145
E146	E145	E146
Q149	Q148	Q149

Supplementary table 2 - Residues composing the epitope of CA1974 Fab.

Residues that form the epitope of Fab CA1974 on human TNFα with equivalent residues on mouse TNFα and cynomolgus monkey TNFα listed for comparison. Non-conserved residues are highlighted in yellow.

	Human TNF **UCB-8733**	Human TNF Human TNFR1 Fab1974 **UCB-8733**
**Data collection**		
Space group	$P\ 2_12_12_1$	$P\ 4_1\ 2_1\ 2$
Cell dimensions		
a, *b*, *c* (Å)	55.12, 82.11, 93.02	99.51, 99.51, 311.35
α, β, γ (°)	90.00, 90.00, 90.00	90.00, 90.00, 90.00
Resolution (Å)	30.0-2.30 (2.36-2.30)	50.00-3.00 (3.08-3.00)
R_{merge}	0.117 (0.674)	0.163 (1.396)
$I / \sigma I$	11.77 (2.61)	13.95 (2.06)
Completeness (%)	98.0 (99.6)	99.9 (100.0)
Redundancy	5.4 (5.1)	12.1 (12.4)
**Refinement**		
Resolution (Å)	27.03-2.30 (2.38-2.30)	49.13-3.00 (3.08-3.00)
No. reflections	18,973 (1,375)	32,445 (2,333)
R_{work} / R_{free}	21.6/26.2 (27.1/33.3)	22.3/25.6 (34.1/36.7)
No. atoms		
Protein	3175	8410
Ligand/ion	41	95
Water	63	20
B-factors (Å^2)		
Protein	29.86	69.69
Ligand/ion	22.28	64.03
Water	25.48	47.65
R.m.s. deviations		
Bond lengths (Å)	0.009	0.003
Bond angles (°)	1.075	0.570

Supplementary table 3 - Data collection and refinement statistics for crystallography.

*A single crystal was used for each structure discussed.

*Values in parentheses are for highest-resolution shell.

#PDB ID: 7KPA [https://www.rcsb.org/structure/7KPA]; 7KPB [https://www.rcsb.org/structure/7KPB]

Supplementary Notes

Supplementary Note 1 – Summary of conditions used for synthesis of UCB-8733

Nomenclature

IUPAC nomenclature was determined with the aid of Biovia draw 2016.

Chemistry

5-{4-Amino-3-[2-(difluoromethoxy)benzylamino]phenyl}pyridin-2(1H)-one was synthesised by known literature methods according to the procedures described in **WO 2013186229**. All reactions were performed under a nitrogen atmosphere using anhydrous solvents and dried glassware.

Analytical Conditions

Analytical LC-MS data were obtained by using the method below.

SQD Mass Spectrometer - ESI Source

Capillary Voltage	0.56 kV
Cone Voltage	55 V
Extractor Voltage	6 V
RF Lens	0.2 V
Source Temperature	150 °C
Desolvation Temperature	350 °C
Desolvation Gas	700 L/Hour
Cone Gas	0 L/Hour
Mass Range	150-650 amu
Scan Time	0.1 seconds

Chromatography

Column	Waters Acquity UPLC BEH C18, 2.1 x 50mm, 1.7 µm
Injection Volume	1-5 µL
UV data	210 to 400 nm
Sample Temperature	Ambient
Column Temperature	40 °C
Flow Rate	1 mL/min
Solvent A2	10 mM Ammonium Formate + 0.1 % Ammonia
Solvent B2	95 % MeCN + 5 % H_2O + 0.1 % Ammonia

Gradient:

Time	%A	%B
0.00	95	5
0.50	95	5
1.75	5	95
2.00	5	95
2.25	95	5

High Resolution MS (HRMS) for final compounds was performed on an Acquity UPLC - Xevo G2 MS.

5-(1-[2-(difluoromethoxy)benzyl]-2-{[3-(2-oxopyrrolidin-1-yl)phenoxy]methyl}-1H-benzimidazol-6-yl)pyridin-2(1H)-one

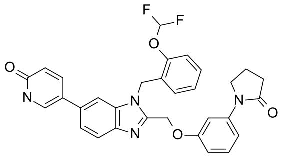

2-[3-(2-oxopyrrolidin-1-yl)phenoxy]acetic acid (1.80 g, 7.7 mmol) and 5-{4-Amino-3-[2-(difluoromethoxy)benzylamino]phenyl}pyridin-2(1H)-one (2.5 g, 7.0 mmol) were dissolved in DCM (70 ml) and N, N'-diisopropylethylamine (1.6 ml, 9.1 mmol) and HATU (3.3 g, 8.4 mmol) were added. The mixture was stirred at room temperature for 30 minutes. DMF (10 ml) was added to solubilise the reaction mixture the solution was stirred at room temperature for 24 h. The solvent partially was evaporated to afford a precipitate which was filtered off, dried then redissolved in acetic acid (10 ml) and heated to 100°C for 4 h. After cooling to room temperature, the solution was basified with $NaHCO_3$ (aqu) and the resulting solid precipitate was filtered, dried then triturated with DCM/MeOH to afford a pale solid (2.8g, 5.0 mmol, 71% yield).

^{1}H-NMR (400 MHz, DMSO-d_6): δ 7.96–7.76 (m, 4H), 7.69 (dd, *J* = 8.6, 1.7 Hz, 1H), 7.50–7.22 (m, 6H), 7.20–7.08 (m, 1H), 7.02 (dd, *J* = 7.8, 1.7 Hz, 1H), 6.78 (ddd, *J* = 8.0, 2.6, 1.2 Hz, 1H), 6.49 (d, *J* = 9.5 Hz, 1H), 5.80 (s, 2H), 5.56 (s, 2H), 3.79 (t, *J* = 7.0 Hz, 2H), 2.54-2.46 (m, coupling obscured by solvent, 2H), 2.12 – 2.00 (m, 2H). ^{13}C NMR (101 MHz, DMSO-d_6): δ 174.5, 162.1, 157.7, 150.6, 149.2 (t, J = 2.9 Hz), 141.2, 140.7, 134.9, 134.0, 133.8, 130.2, 130.0, 129.0, 125.9, 125.7, 123.1, 120.5, 118.1, 118.0, 117.6, 116.9 (t, J = 258 Hz), 113.2, 110.0, 109.0, 106.9, 62.1, 48.6, 43.9, 32.9, 17.8. LCMS (ESI+) (m/z) 557 $(MH)^+$ 1.33 min. HRMS (*m/z*): [MH]+ calcd for $C_{31}H_{27}F_2N_4O_4$ 557.2000; found 557.2014.

Supplementary References

1. McMillan, D. et al. Structural insights into disruption of TNF-TNFR1 signalling by small molecules stabilising a distorted TNF. Nat Commun, in press (2021)
2. O'Connell, J. et al. Small molecules that inhibit TNF signalling by stabilising an asymmetric form of the trimer. Nat Commun 10, 5795 (2019).
