# Supplementary material for: A conformation-selective monoclonal antibody against a small molecule-stabilised signalling-deficient form of TNF
Source: Nat Commun. 2021 Jan 25;12:583. doi: 10.1038/s41467-020-20825-6 (PMC7835358; doi:10.1038/s41467-020-20825-6)
Supplement: Supplementary file 2 — Reporting Summary [file 41467_2020_20825_MOESM2_ESM.pdf]

nature research

NCOMMS-17-33690C

Corresponding author(s): Daniel Lightwood

Last updated by author(s): Nov 30, 2020

Reporting Summary

Nature Research wishes to improve the reproducibility of the work that we publish. This form provides structure for consistency and transparency in reporting. For further information on Nature Research policies, see our Editorial Policies and the Editorial Policy Checklist.

Statistics

For all statistical analyses, confirm that the following items are present in the figure legend, table legend, main text, or Methods section.

n/a	Confirmed	
☐	☒	The exact sample size (n) for each experimental group/condition, given as a discrete number and unit of measurement
☐	☒	A statement on whether measurements were taken from distinct samples or whether the same sample was measured repeatedly
☐	☒	The statistical test(s) used AND whether they are one- or two-sided<br>*Only common tests should be described solely by name; describe more complex techniques in the Methods section.*
☒	☐	A description of all covariates tested
☐	☒	A description of any assumptions or corrections, such as tests of normality and adjustment for multiple comparisons
☐	☒	A full description of the statistical parameters including central tendency (e.g. means) or other basic estimates (e.g. regression coefficient) AND variation (e.g. standard deviation) or associated estimates of uncertainty (e.g. confidence intervals)
☒	☐	For null hypothesis testing, the test statistic (e.g. F, t, r) with confidence intervals, effect sizes, degrees of freedom and P value noted<br>*Give P values as exact values whenever suitable.*
☒	☐	For Bayesian analysis, information on the choice of priors and Markov chain Monte Carlo settings
☒	☐	For hierarchical and complex designs, identification of the appropriate level for tests and full reporting of outcomes
☒	☐	Estimates of effect sizes (e.g. Cohen's d, Pearson's r), indicating how they were calculated

Our web collection on statistics for biologists contains articles on many of the points above.

Software and code

Policy information about availability of computer code

Data collection	Experiments at the Advance Photon Source (APS) were run on custom software for LS-CAT beamlines 21-ID-F and 21-ID-G
Data analysis	GE Healthcare T200 Evaluation software (version 1.0)<br>Thermo Multiskan EX plate reader with Ascent Software version 2.6<br>Microsoft Excel<br>BD Biosciences FlowJo software<br><br>Applied published crystallography tools:<br><br>Data reduction - XDS (Versions December 31, 2011 and October 15, 2015)<br>Data scaling - XSCALE (Versions December 31, 2011 and October 15, 2015)<br>Refinement - PHENIX software suite (dev_2443)<br>Refinement - CCP4 program suite, Refmac5 (Version 5.7.0029)<br>Validation - Molprobity<br>Validation - wwPDB validation service<br><br>Statistical tools<br>SAS 9.4, SAS Institute Inc., Cary, NC, USA

For manuscripts utilizing custom algorithms or software that are central to the research but not yet described in published literature, software must be made available to editors and reviewers. We strongly encourage code deposition in a community repository (e.g. GitHub). See the Nature Research guidelines for submitting code & software for further information.

Data

Policy information about availability of data

All manuscripts must include a data availability statement. This statement should provide the following information, where applicable:

- Accession codes, unique identifiers, or web links for publicly available datasets
- A list of figures that have associated raw data
- A description of any restrictions on data availability

Relevant datasets generated during and/or analysed during the current study are provided in a source data file. The source data underlying Figs 1, 2, 6, supplementary Figs 1, 2, 7, 8 and 10 are provided.
PDB codes for the novel structures in this paper are 7KPA and 7KPB

Field-specific reporting

Please select the one below that is the best fit for your research. If you are not sure, read the appropriate sections before making your selection.

☒ Life sciences ☐ Behavioural & social sciences ☐ Ecological, evolutionary & environmental sciences

For a reference copy of the document with all sections, see nature.com/documents/nr-reporting-summary-flat.pdf

Life sciences study design

All studies must disclose on these points even when the disclosure is negative.

Sample size	no sample size calculation was made in the current study. some data consists of n=2 independent experiments whereas other data are generated from representative n=1 experiments.
Data exclusions	no data was excluded
Replication	where applied, all attempts at replication were successful and are included in the data analyses
Randomization	randomization was not relevant to the current study
Blinding	blinding was not relevant to the current study

Reporting for specific materials, systems and methods

We require information from authors about some types of materials, experimental systems and methods used in many studies. Here, indicate whether each material, system or method listed is relevant to your study. If you are not sure if a list item applies to your research, read the appropriate section before selecting a response.

Materials & experimental systems

n/a	Involved in the study	
☐	☒	Antibodies
☐	☒	Eukaryotic cell lines
☒	☐	Palaeontology and archaeology
☐	☒	Animals and other organisms
☒	☐	Human research participants
☒	☐	Clinical data
☒	☐	Dual use research of concern

Methods

n/a	Involved in the study	
☒	☐	ChIP-seq
☐	☒	Flow cytometry
☒	☐	MRI-based neuroimaging

Antibodies

Antibodies used

biotinylated goat F(ab)2 anti-human Fc reagent (Jackson ImmunoResearch, catalogue number 109-066-098)

HRP-conjugated goat anti-mouse IgG Fcγ-specific antibody (Jackson ImmunoResearch, catalogue number 115-036-071)

goat anti-mouse IgG F(ab)2-specific antibody (Jackson ImmunoResearch, catalogue number 115-036-072)

Goat anti-mouse IgG Fcγ-specific antibody (Jackson ImmunoResearch, catalogue number 115-006-071)

anti-mouse-Alexa fluor-488 antibody (Jackson immunoResearch, catalogue number 115-545-071)

biotinylated anti-TNFα detection antibody (R&D Systems, catalogue number BAF210)

an anti-human TNFR1 monoclonal antibody at 10 µg/ml (R & D Systems, catalogue number MAB225)

Goat-anti-mouse-Alexa488 secondary antibody (Molecular Probes, catalogue number A-11001)

Validation

data associated with validation of the above antibodies can be found on the manufacturers' websites.

For anti-TNFa antibody (RnD systems, BAF210) the following references are cited:
1.Idriss, H.T. and J.H. Naismith (2000) Microsc. Res. Tech. 50:184.
2.Hehlgans, T. and K. Pfeffer (2005) Immunology 115:1.
3.Pennica, D. et al. (1984) Nature 312:724.
4.Tang, P. et al. (1996) Biochemistry 35:8216.
5.Eissner G. et al. (2004) Cytokine Growth Factor Rev. 15:353.
6.Black, R.A. et al. (1997) Nature 385:729.
7.Moss, M.L. et al. (1997) Nature 385:733.
8.Loetscher, H. et al. (1991) J. Biol. Chem. 266:18324.
9.Clark, I.A. (2007) Cytokine Growth Factor Rev. 18:335.
10.Romanatto, T. et al. (2007) Peptides 28:1050.
11.Hector, J. et al. (2007) Horm. Metab. Res. 39:250.

For anti-TNFR1 monoclonal antibody (RnD systems, MAB225) the following references are cited:
1 Human TNF-Luc reporter mouse: A new model to quantify inflammatory responses
Authors: F Minshawi, MRH White, W Muller, N Humphreys, D Jackson, BJ Campbell, A Adamson, S Papoutsopo
Sci Rep, 2019;9(1):193.
Species: Human
Sample Types: Whole Cells
Applications: Neutralization
2 Low doses of LPS exacerbate the inflammatory response and trigger death on TLR3-primed human monocytes
Authors: M Monguió-To, M Franquesa, MR Sarrias, FE Borràs
Cell Death Dis, 2018;9(5):499.
Species: Human
Sample Types: Whole Cells
Applications: Neutralization
3 Bispecific T cell engager (BiTEï¿½) antibody constructs can mediate bystander tumor cell killing
Authors: SL Ross, M Sherman, PL McElroy, JA Lofgren, G Moody, PA Baeuerle, A Coxon, T Arvedson
PLoS ONE, 2017;12(8):e0183390.
Species: Human
Sample Types: Whole Cells
Applications: Neutralization
4 ST6Gal-I sialyltransferase promotes chemoresistance in pancreatic ductal adenocarcinoma by abrogating gemcitabine-mediated DNA damage
Authors: A Chakrabort, KA Dorsett, HQ Trummell, ES Yang, PG Oliver, JA Bonner, DJ Buchsbaum, SL Bellis
J. Biol. Chem., 2017;0(0):.
Species: Human
Sample Types: Whole Cells
Applications: Neutralization
5 ST6Gal-I sialyltransferase promotes tumor necrosis factor (TNF)-mediated cancer cell survival via sialylation of the TNF receptor 1 (TNFR1) death receptor
Authors: AT Holdbrooks, CM Britain, SL Bellis
J. Biol. Chem., 2017;0(0):.
Species: Human
Sample Types: Whole Cells
Applications: Neutralization
6 TNF? promotes CAR-dependent migration of leukocytes across epithelial monolayers
Sci Rep, 2016;6(0):26321.
Species: Human
Sample Types: Whole Cells
Applications: Neutralization
7 TNF? promotes CAR-dependent migration of leukocytes across epithelial monolayers
Sci Rep, 2016;6(0):26321.
Species: Human
Sample Types: Whole Cells
Applications: Neutralization
8 Circulating TNF receptors 1 and 2 are associated with the severity of renal interstitial fibrosis in IgA nephropathy.

Authors: Sonoda Y, Gohda T, Suzuki Y, Omote K, Ishizaka M, Matsuoka J, Tomino Y
PLoS ONE, 2015;10(4):e0122212.
Species: Human
Sample Types: Whole Tissue
Applications: IHC
9 Enhancement of TWIK-related acid-sensitive potassium channel 3 (TASK3) two-pore domain potassium channel activity by tumor necrosis factor alpha.
Authors: El Hachmane M, Rees K, Veale E, Sumbayev V, Mathie A
J Biol Chem, 2014;289(3):1388-401.
Species: Human
Sample Types: Whole Cells
Applications: Neutralization
10 Apoptotic signaling through Fas and TNF receptors ameliorates GVHD in mobilized peripheral blood grafts.
Authors: Mizrahi K, Yaniv I, Ash S, Stein J, Askenasy N
Bone Marrow Transplant, 2014;49(5):640-8.
Species: Human
Sample Types: Whole Cells
Applications: Flow Cytometry

11 Tumor necrosis factor stimulates osteoclastogenesis from human bone marrow cells under hypoxic conditions.
Authors: Nomura T, Aoyama M, Waguri-Nagaya Y, Goto Y, Suzuki M, Miyazawa K, Asai K, Goto S
Exp Cell Res, 2014;321(2):167-77.
Species: Human
Sample Types: Whole Cells
Applications: Neutralization
12 High efficiency cell-specific targeting of cytokine activity.
Authors: Garcin G, Paul F, Staufenbiel M, Bordat Y, Van der Heyden J, Wilmes S, Cartron G, Apparailly F, De Koker S, Piehler J, Tavernier J, Uze G
Nat Commun, 2014;5(0):3016.
Species: Mouse
Sample Types: Whole Cells
Applications: Flow Cytometry
13 The transmembrane domains of TNF-related apoptosis-inducing ligand (TRAIL) receptors 1 and 2 co-regulate apoptotic signaling capacity.
Authors: Neumann S, Bidon T, Branschadel M, Krippner-Heidenreich A, Scheurich P, Doszczak M
PLoS ONE, 2012;7(8):e42526.
Species: Mouse
Sample Types: Whole Cells
Applications: Flow Cytometry
14 Tumour necrosis factor alpha stimulates the production of monocyte chemoattractants by extravillous trophoblast cells via differential activation of MAPK pathways.
Authors: Renaud SJ, Sullivan R, Graham CH
Placenta, 2009;30(4):313-9.
Species: Human
Sample Types: Whole Cells
Applications: Flow Cytometry
15 Der p 1 suppresses indoleamine 2, 3-dioxygenase in dendritic cells from house dust mite-sensitive patients with asthma.
Authors: Maneechotesuwan K, Wamanuttajinda V, Kasetsinsombat K, Huabprasert S, Yaikwawong M, Barnes PJ, Wongkajornsilp A
J. Allergy Clin. Immunol., 2009;123(1):239-48.
Species: Human
Sample Types: Whole Cells
Applications: Flow Cytometry
16 IL-6 cytoprotection in hyperoxic acute lung injury occurs via suppressor of cytokine signaling-1-induced apoptosis signal-regulating kinase-1 degradation.
Authors: Kolliputi N, Waxman AB
Am. J. Respir. Cell Mol. Biol., 2009;40(3):314-24.
Species: Human
Sample Types: Cell Lysates
Applications: Western Blot
17 Differential responses of FLIPLong and FLIPShort-overexpressing human myeloid leukemia cells to TNF-alpha and TRAIL-initiated apoptotic signals.
Authors: Seal S, Hockenbery DM, Spaulding EY, Kiem HP, Abbassi N, Deeg HJ
Exp. Hematol., 2008;36(12):1660-72.
Species: Human
Sample Types: Whole Cells
Applications: Flow Cytometry
18 Death receptor expression is associated with poor response to chemotherapy and shorter survival in metastatic ovarian carcinoma.
Authors: Dong HP, Kleinberg L, Silins I, Florenes VA, Trope CG, Risberg B, Nesland JM, Davidson B

Cancer, 2008;112(1):84-93.
Species: Human
Sample Types: Whole Cells
Applications: Flow Cytometry
19 A novel mechanism of CD40-induced apoptosis of carcinoma cells involving TRAF3 and JNK/AP-1 activation.
Authors: Georgopoulos NT, Steele LP, Thomson MJ, Selby PJ, Southgate J, Trejdosiewicz LK
Cell Death Differ., 2006;13(10):1789-801.
Species: Human
Sample Types: Whole Cells
Applications: Flow Cytometry
20 Human intestinal intraepithelial lymphocytes keep TNF alpha levels low by cell uptake and feedback inhibition of transcription.
Authors: Ebert EC, Mehta V
Cell. Immunol., 2006;241(1):7-13.
Species: Human
Sample Types: Whole Cells
Applications: Flow Cytometry
21 A role for tumor necrosis factor alpha in death of dopaminergic neurons following neural transplantation.
Authors: Clarke DJ, Branton RL
Exp. Neurol., 2002;176(1):154-62.
Species: Rat
Sample Types: Whole Cells
Applications: Neutralization
22 Negative selection by apoptosis enriches progenitors in naive and expanded human umbilical cord blood grafts.
Authors: Mizrahi K, Ash S, Peled T, Yaniv I, Stein J, Askenasy N
Bone Marrow Transplant, 0;49(7):942-9.
Species: Human
Sample Types: Whole Cells
Applications: Flow Cytometry

Eukaryotic cell lines

Policy information about cell lines

Cell line source(s)	Human embryonic kidney (HEK) Jump In cells (Thermo Fisher, catalogue number A15008)<br><br>Tni cells (Expression Systems, LLC, catalogue number 94-002S)
Authentication	flow cytometry was used to authenticate the level of expression of TNFR1 on HEK Jump In cells. details of which are shown in supplementary figure 1.
Mycoplasma contamination	all cell lines were negative for mycoplasma
Commonly misidentified lines<br>(See ICLAC register)	N/A

Animals and other organisms

Policy information about studies involving animals; ARRIVE guidelines recommended for reporting animal research

Laboratory animals	Female Sprague Dawley rats
Wild animals	N/A
Field-collected samples	N/A
Ethics oversight	Approval for use of animals for immunisation was provided through the UCB Pharma, UK Animal Welfare and Ethical Review Body (AWERB) and the license was granted by the UK Home Office. At the end of the study the mice were anesthetised with isoflurane, terminal bleeds taken and then sacrificed using a Schedule 1 method in accordance with the Animals Scientific Procedures Act (ASPA).

Note that full information on the approval of the study protocol must also be provided in the manuscript.

Flow Cytometry

Plots

Confirm that:

☒ The axis labels state the marker and fluorochrome used (e.g. CD4-FITC).

☒ The axis scales are clearly visible. Include numbers along axes only for bottom left plot of group (a 'group' is an analysis of identical markers).

☒ All plots are contour plots with outliers or pseudocolor plots.

☒ A numerical value for number of cells or percentage (with statistics) is provided.

Methodology

Sample preparation	Purified recombinant CA1974 IgG was tested for binding to TNFα-small molecule inhibitor complex in a flow cytometry assay using human embryonic kidney (HEK) Jump In cells (Thermo Fisher, catalogue number A15008), which overexpress TNFR1 after induction with doxycycline at 1 µg/ml for 2.5 hours (supplementary Fig. 1a). HEK cells were trypsinised and incubated for 2 h in medium to allow recovery of digested TNFR1 levels. Human TNFα at a range of concentrations was pre-incubated with 4.0 µM UCB-9260 or 0.4% DMSO (equivalent concentration of DMSO) for 1 h at 37 °C. The pre-incubation mix was added to a 3-fold v/v excess of the cells giving a TNFα concentration of either 25 or 250 ng/ml. The sample was incubated for 1 h on ice. Cells were washed, fixed (1.5% PFA) and stained with 10 µg/ml CA1974 antibody for 1h on ice. After washing again, binding was revealed using a secondary anti-mouse-Alexa fluor-488 antibody (Jackson immunoResearch, catalogue number 115-545-071). Samples were run on a BD Biosciences Canto II Flow Cytometer with 3 lasers and a standard 4:2:2 configuration. The Alexa fluor-488 was excited by a 488 nm laser and fluorescence collected through the 530/30 BP filter. Gating of single viable cells (excluding doublets and other cell debris) was made using a forward scatter versus side scatter plot (supplementary Fig. 1b). TNFR1 expression within the population of gated cells following doxycycline at 1 µg/ml for 2.5 hours was assessed in a separate experiment using an anti-human TNFR1 monoclonal antibody at 10 µg/ml (R & D Systems, catalogue number MAB225) followed by staining with Goat-anti-mouse-Alexa488 secondary antibody (Molecular Probes, catalogue number A-11001) at 1:200 dilution. This showed heterogeneous expression of TNFR1 as well as the presence of a population which appeared negative for receptor consistent with staining observed with CA1974 (supplementary Fig. 1a).
Instrument	BD Biosciences Canto II Flow Cytometer with 3 lasers and a standard 4:2:2 configuration
Software	BD Biosciences FlowJo software
Cell population abundance	N/A
Gating strategy	Gating of single viable cells (excluding doublets and other cell debris) was made using a forward scatter versus side scatter plot (supplementary Fig. 1b)

☒ Tick this box to confirm that a figure exemplifying the gating strategy is provided in the Supplementary Information.
